# Diagnostic Value and Application of Infrared Thermography in the Analysis of Circumanal Gland Tumors

**DOI:** 10.3389/fvets.2021.692221

**Published:** 2021-07-27

**Authors:** Erika B. M. Zanuto, Samanta R. Melo, Eric V. Januário, Gustavo A. A. L. Fernandes, Julia M. Matera

**Affiliations:** ^1^Department of Surgery, School of Veterinary Medicine and Animal Science, University of São Paulo, São Paulo, Brazil; ^2^São Paulo School of Business Administration—Getúlio Vargas Foundation, São Paulo, Brazil

**Keywords:** diagnosis, oncology, thermography, diagnostic, dogs

## Abstract

In dogs, circumanal tumors are the third most common skin neoplasm. Circumanal gland adenomas (CAGAs) have a good prognosis. Contrastingly, circumanal gland adenocarcinomas (CAGAC) have high relapse rates and may be metastatic. This study aimed to investigate the utility of thermal imaging as an ancillary modality for the diagnosis of canine CAGA and CAGAC. We analyzed the following parameters: SpT, temperature measured at the tumor center; SpNT, temperature measured at a healthy sphincter skin spot distant from the tumor; TA, temperature measured at a tumor-encompassing ellipse-shaped area; and NTA, temperature measured at an ellipse-shaped area of the healthy sphincter skin distant from the tumor. In CAGAs, the mean SpT and SpNT temperature values differed by −1.45°C (*p* < 0.01) while the mean TA and NTA temperature values differed by −0.96°C (*p* < 0.05). In CAGACs, mean SpT and SpNT temperatures differed by −1.71°C (*p* < 0.01) while the mean TA and NTA temperatures differed by −1.69°C (*p* < 0.01). The mean SpT and TA temperature values measured in CAGAs and CAGACs differed by −0.10°C (*p* = 0.87) and 0.52°C (*p* = 0.38), respectively. Both tumors were colder than healthy sphincter skin. However, a substantial number of CAGACs were colder than CAGAs. Temperature differences ≥ 1°C between tumors and healthy sphincter skin increased the probability of CAGAC diagnosis by 17.45%. Thermal imaging allowed discrimination between healthy and tumoral tissues; therefore, it could be a good ancillary diagnostic modality.

## Introduction

Circumanal tumors, which are also termed as perianal or hepatoid tumors, are common neoplasms in dogs that account for 5.8–13.5% of skin tumors in this species (1–3). Adenomas are the most common skin tumors (46–96%), followed by adenomas (3–54%) (4–9).

Circumanal tumors are mostly benign. However, there are inconsistent reports regarding their incidence and prevalence, which suggests the need for further research on these neoplasms (6–9), including diagnostic and therapeutic protocols. Surgical resection is the first-line treatment choice for circumanal tumors (10). Malignant neoplasms require wider resection margins; moreover, surgeons must consider the tumor type for appropriate surgical planning. Although safety margins are important for preventing recurrence, functional properties such as external anal sphincter contraction should be preserved to avoid permanent fecal incontinence. Therefore, there is a need for further research on complementary diagnostic methods.

Thermal imaging is an ancillary diagnostic method for neoplastic diseases. It is based on the premise that enhanced blood flow to neoplastic and adjacent tissues, as well as accompanying increased tumorigenesis and higher metabolic rates, cause temperature elevation in affected tissues compared with normal tissues (11). Further, chronic inflammation associated with solid tumors may induce temperature changes in neoplastic tissues (12).

Increased temperatures may be detected in peritumoral areas, even with the affected area being colder than normal skin. Mechanisms underlying these temperature changes remain unclear; however, they are thought to reflect peripheral neoangiogenesis (12). Konerding et al. (13) reported no temperature elevation in human neoplasms transplanted into nude mice, which were either colder or of similar temperature compared with healthy skin. The mechanisms underlying this thermal behavior remain unclear. Nonetheless, functional impairment of newly formed vessels may reduce blood flow in tumoral tissues compared with normal tissues (14).

Thermal imaging cameras measure infrared radiation emitted from the skin. Under appropriate environmental conditions, these cameras can establish a precise ratio between the skin surface temperature and infrared radiation. Thermal radiation patterns are converted into images depicting the skin surface temperature distribution (15). Modern cameras can detect temperature variations of <0.1°C per square millimeter of tissue (16).

In medicine, thermal imaging is a non-invasive, safe, and fast diagnostic modality that has been used for complementary diagnosis, prognostication, and follow-up of several neoplastic diseases (17). Several studies on breast cancer in women have reported surface temperature elevations of up to 3°C around the neoplasms. Moreover, thermal imaging allows early diagnosis and provides significant prognostic information (16–22).

However, inherent limitations impede the use of thermal imaging as a standalone diagnostic tool, including the high false-positive rates, intermediate sensitivity, and inability to measure deep tissue temperatures. Nonetheless, the reliability of these methods has been increased by advanced software development, mathematical probability studies, and enhanced image quality (23, 24).

Medically, the use of thermal imaging is not limited to oncology and has been applied in sports medicine, orthopedics, neurology, anesthesiology and pain management, angiology, and animal and human plastic reconstructive surgery (25–30).

There is increasing popularity of thermal imaging in veterinary oncology. Pavelski et al. (31) reported that canine mammary tumors are significantly warmer than healthy glands, regardless of the tumor size and location. Melo et al. (29) reported that thermal imaging was a promising ancillary modality for mast cell tumor diagnosis. This study aimed to determine the utility of thermal imaging as an ancillary modality for the diagnosis of circumanal gland adenomas (CAGAs) and circumanal gland adenocarcinomas (CAGAC) in dogs.

## Materials and Methods

### Animal Selection

The animal study was reviewed and approved by Ethics Committee of the School of Veterinary Medicine and Animal Science, University of São Paulo. Written informed consent was obtained from the owners for the participation of their animals in this study. We included 44 male and 7 female dogs [age: 15.89 (8 months–18 years) years] dogs seen at the Small Animal Surgery Department of the Veterinary Hospital of the School of Veterinary Medicine and Animal Science of the University of São Paulo between August 2015 and December 2018. The average weight and rectal temperature were 15.89 [4.40–36.00] kg and 38.68 [37.70–39.8] °C, respectively (Table 1). This study included male and female dogs presenting with perianal neoplasms diagnosed with CAGA or CAGAC based on cytologic and histologic findings.

**Table 1:** Temperature values obtained through thermographic examination of each animal with their respective histopathological examinations, ages, weights, and rectal temperatures.

**Dog**	**Tumor**	**Age** **(year)**	**Weight** **(kg)**	**Rectal Temperature** **(** **°** **C)**	**Spt** **(** **°** **C)**	**SpNT** **(** **°** **C)**	**AT** **(** **°** **C)**	**ANT** **(** **°** **C)**
A1	CAGAG	11	13	38.4	32.1	37.3	33.4	37.2
A2	CAGAG	13			35.5	36.8	35	36.7
A3	CAGA	11	33.85	39.8	37.6	37.8	37.8	37.8
A4	CAGA	12	21.2	38.3	37.7	37.5	38.3	37.6
A5	CAGA	13	10		26.7	34.1	29	32.6
A6	CAGAG	14	30	38.8	37.3	37.3	36.9	37.2
A7	CAGA	12	7.5	38.9	38.1	37.6	38	37.7
A8	CAGA	0.75	31	38.9	38.5	37.4	38.3	38.4
A9	CAGA	12	7.2		34.5	34.3	35.3	34.3
A10	CAGA	11	14	37.9	36.3	37.4	36.7	37.4
A11	CAGAG	9	15.3	39.7	37.4	36.3	37.3	35.9
A12	CAGA	10	17.8	39.5	34.5	33.8	34.7	34
A13	CAGA	9	5.1	39	36	38.2	35.8	37.1
A14	CAGA	11	19.8		38.2	38.3	38.5	38.5
A15	CAGA	12	28.4		37.8	37.7	37.8	37.8
A16	CAGA	13	27.8	39.5	34.7	38.6	36	38.1
A17	CAGAG	13	23.8	38.3	36.4	35.2	36.2	35.1
A18	CAGAG	14	9.45	39.1	33.3	36.9	32.3	36.8
A19	CAGAG	10	36		36.2	37	32.1	35.8
A20	CAGAG	14	13.8		34.6	38.7	36.3	37.7
A21	CAGA	18	10.4		29.7	35.3	31.1	35.2
A22	CAGA	11	15	37.9	36.8	35.7	36.3	36.7
A23	CAGAG	12	4.95		37	35.4	36.2	35.2
A24	CAGAG	9			36.6	37	36.5	35.7
A25	CAGAG	6			34.5	36.7	35.2	36.7
A26	CAGA	11			32.7	31.6	32.9	31.9
A27	CAGAG	12	10.5		32.1	37.2	33	37
A28	CAGA	13	25.3	39.7	36.4	38.4	36.8	38.1
A29	CAGAG	11	15	38	36.3	36.7	36.2	36.5
A30	CAGAG	12	9	38.2	31.1	36.5	32.8	36.3
A31	CAGA	10	9.65	38.1	33.8	36.9	34.2	36.8
A32	CAGAG	11	10.7	38.3	37.2	37.4	35.7	37
A33	CAGA	14	15.6	38.6	37.6	38.1	37.6	38.3
A34	CAGA	16	4.4	38.4	33.7	34.9	33.9	34.7
A35	CAGA	13	13	38.2	30.4	36.5	32.4	36.6
A36	CAGAG	13	13.25	39.1	35.9	34.3	36	34.5
A37	CAGA	11	9.3		37.5	38.5	37.7	38.4
A38	CAGA	9	8.2		35.9	35.6	36	35.5
A39	CAGA	16	5.2	37	33	36.2	32.9	36.5
A40	CAGA	12	7.3		32.7	35.1	32.8	35.2
A41	CAGA	13	7.8	38.6	28.5	34.6	29.8	34.4
A42	CAGA	12	14.35	38.7	34	36.7	35	36.6
A43	CAGAG	13	17	38.7	34.6	36.3	34.3	35.8
A44	CAGAG	11	21.5	38.3	37.2	37.3	35.3	37.5
A45	CAGA	8	15		38.5	40.2	37.7	37.9
A46	CAGAG	13	31.6		32.9	37.2	33.5	37
A47	CAGAG	13	6.45		33.5	36.7	33.2	36.8
A48	CAGAG	11	8.9	39	37.6	37.7	37.5	37.6
A49	CAGA	17			36.2	37.4	36.6	37.3
A50	CAGA	10	29	39.3	37.2	37.5	37.7	37.6
A51	CAGA	11	27.6	39.1	35.8	35.7	36	35.8

*CAGA, circumanal gland adenoma; CAGAC, circumanal gland adenocarcinoma; SpT, temperature measured in the center of circumanal tumors; SpNT—temperature measured in a healthy sphincter skin spot located as far from the tumor as possible; TA—ellipse-shaped area encompassing the tumor; NTA—ellipse-shaped area of healthy sphincter skin located as far from the tumor as possible*.

### Obtaining Thermographic Images

**Figure 1:**
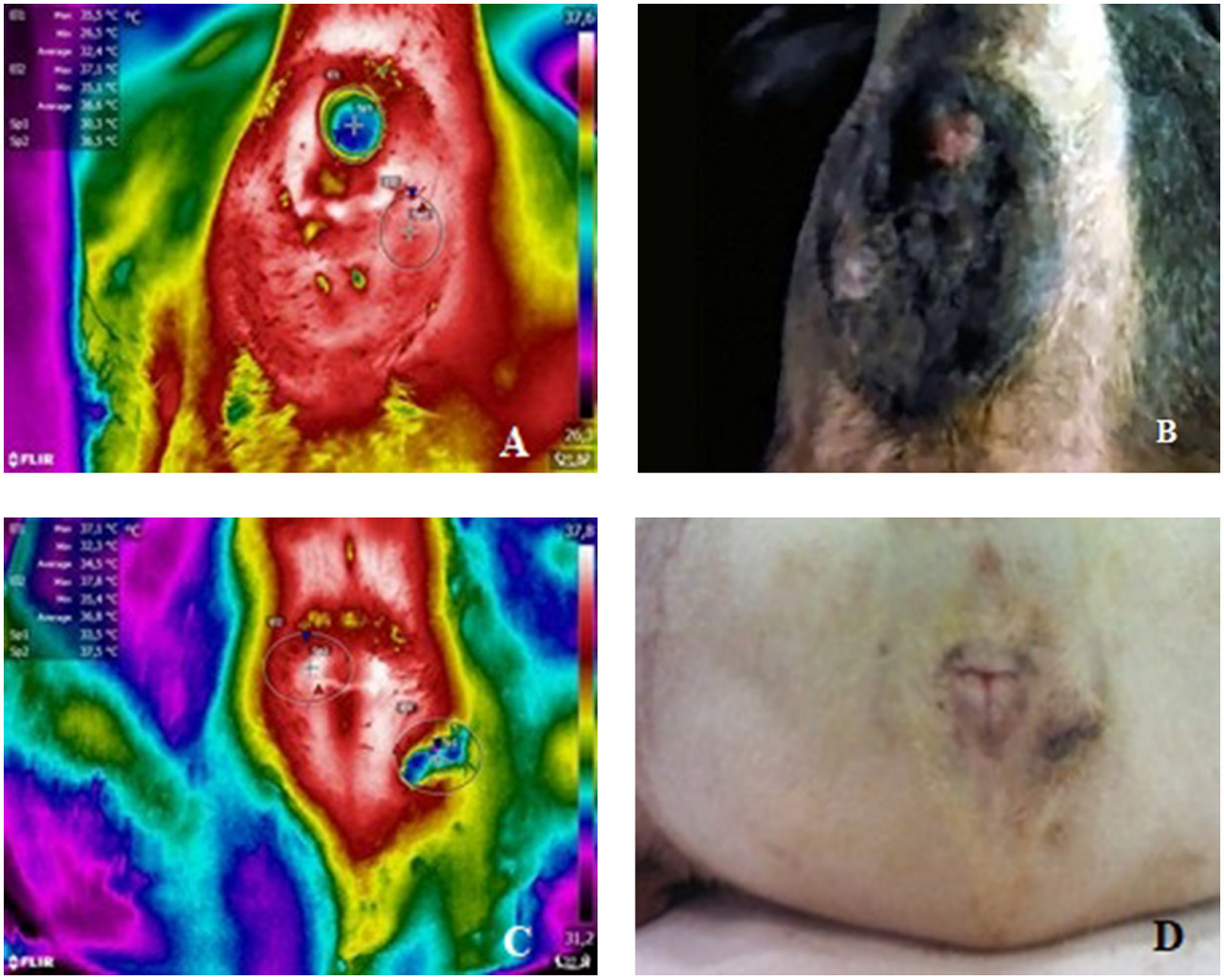
**(A)** Thermal image of patient **50** (CAGA) depicting SpT (Sp1), SpNT (Sp2), TA (E1), and NTA (E2) temperature readings. **(B)** Corresponding (non-thermal) image. **(C)** Thermal image of patient **28** (CAGAC) depicting SpT (Sp1), SpNT (Sp2), TA (E1), and NTA (E2) temperature readings. **(D)** Corresponding (non-thermal) image.

Thermal imaging of the perianal region was conducted using a FLIR® T650sc camera (Wi-Fi thermal imaging camera with a resolution of 307,000 pixels and sensitivity of 30 mK). Temperature measurements were performed inside the surgical theater. Further, the temperature was regulated by air conditioning with variations <1°C during image acquisition. The temperature and relative humidity of the environment were measured using a digital hygrometer connected with the Flir ResearchIR® Software, which automatically performs variation homogenization for further image analysis. Perianal tumor images were acquired before surgical resection, within 15 min after hair clipping, and immediately after anesthetic induction. There were no anesthesia-induced body temperature changes. As shown in Figure 1, all patients were placed in the prone position on the operating table with the pelvic limbs flexed. All images were acquired with the camera positioned at the same distance (0.5 m) from the patient and under similarly rigorous conditions.

### Image Analysis

We analyzed the following parameters from the thermographic images:

- Center of the circumanal tumor (tumoral spot, SpT);
- Healthy sphincter skin spot distant from the tumor (non-tumoral spot, SpNT);
- Ellipse-shaped area encompassing the tumor (tumoral area, TA);
- Ellipse-shaped area of healthy sphincter skin distant from the tumor (non-tumoral area, NTA)

### Statistical Analysis

The mean temperatures of CAGAs, CAGACs, and healthy sphincter skin were compared using Student's *t*-test. We accounted for different numbers of observations per group and potential heteroscedasticity. Tests were performed as described by Acock (32). Statistical analyses were performed using Stata software version 14.

To investigate the relationship between tumor-based temperature differences, the following equation was estimated using probit regression with robust estimators (33):

$$\begin{array}{l} \Phi_{i}=\beta_{0}+\beta_{1}.\left(SpNT-SpT\right)+\beta_{2}.\left(NTA-TA\right)+\varepsilon \end{array}$$

Where Φ_*i*_ is a tumor type indicator (1 = adenocarcinoma; 0 = adenoma); SpT is the temperature at the tumoral spot; and SpNT is the temperature at the non-tumoral spot. TA and NTA stand for average tumoral and non-tumoral areas, respectively.

## Results

Perianal thermographic assessment was performed in 31 and 20 dogs with CAGA and CAGAC, respectively. Temperature data were collected from the SpT, SpNT, TA, and NTA. Table 1 shows the thermal imaging findings in circumanal gland tumors. Figure 1 shows thermal images displaying SpT, SpNT, TA, and NTA temperature readings and the corresponding non-thermal images.

Mean SpT and SpNT temperatures recorded in CAGAs (35.06 °C and 36.51°C) and CAGACs (35.17 and 36.88°C) differed by −1.45°C (*p* < 0.01) and −1.71°C (*p* < 0.01), respectively. Further, mean TA and NTA temperatures recorded in CAGAs (35.47 and 36.42°C) and CAGACs (34.95 and 36.57°C) differed by −0.96°C (*p* < 0.05) and −1.69°C (*p* < 0.01), respectively. Taken together, CAGAs and CAGACs were colder than healthy sphincter skin. Further, the mean SpT and TA temperatures recorded in CAGAs and CAGACs differed by −0.10°C (*p* = 0.87) and 0.52°C (*p* = 0.38), respectively.

**Figure 2:**
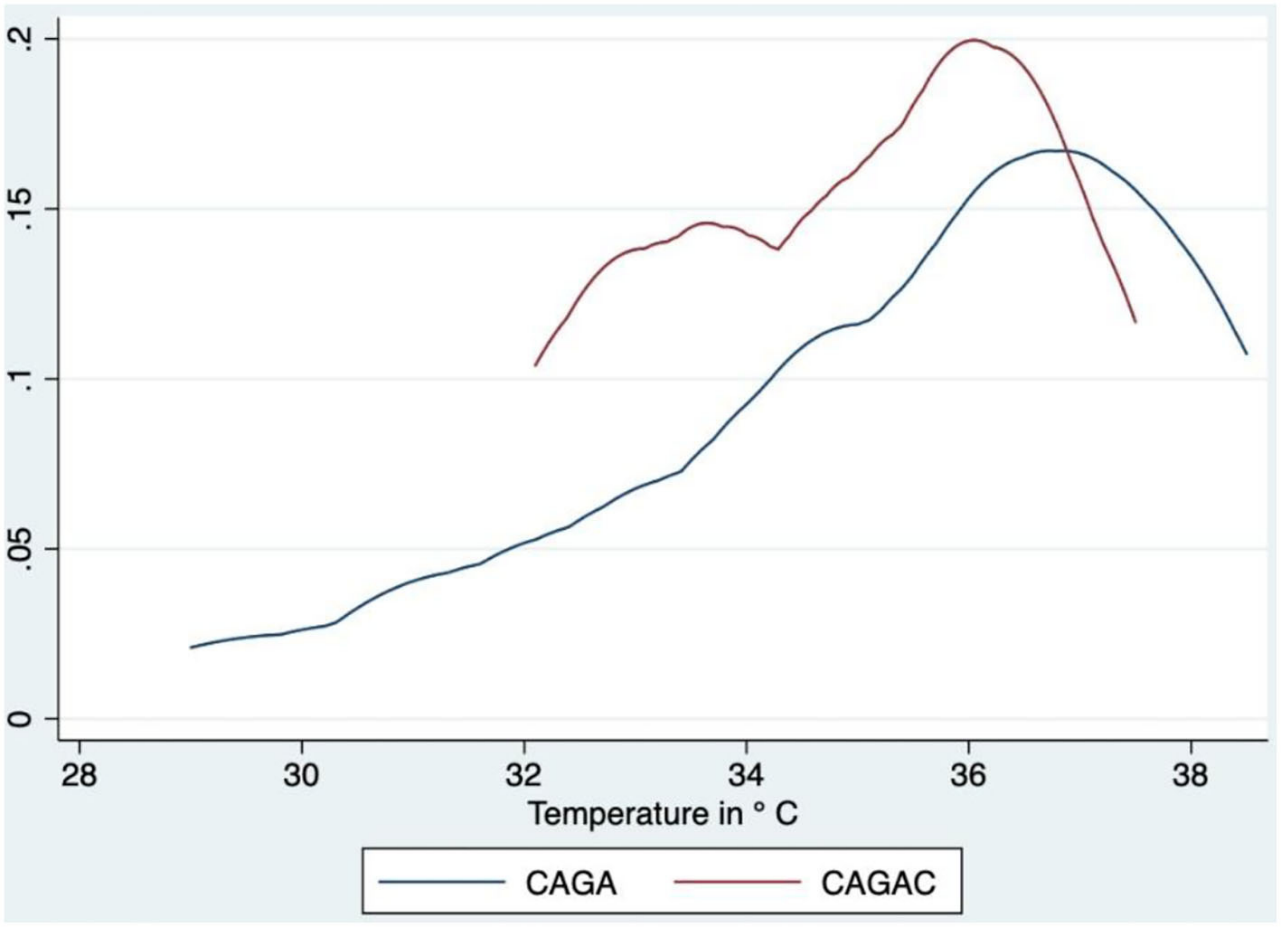
Comparative analysis of probability density functions—TA. CAGA, circumanal gland adenoma; CAGAC, circumanal gland adenocarcinoma.

**Figure 3:**
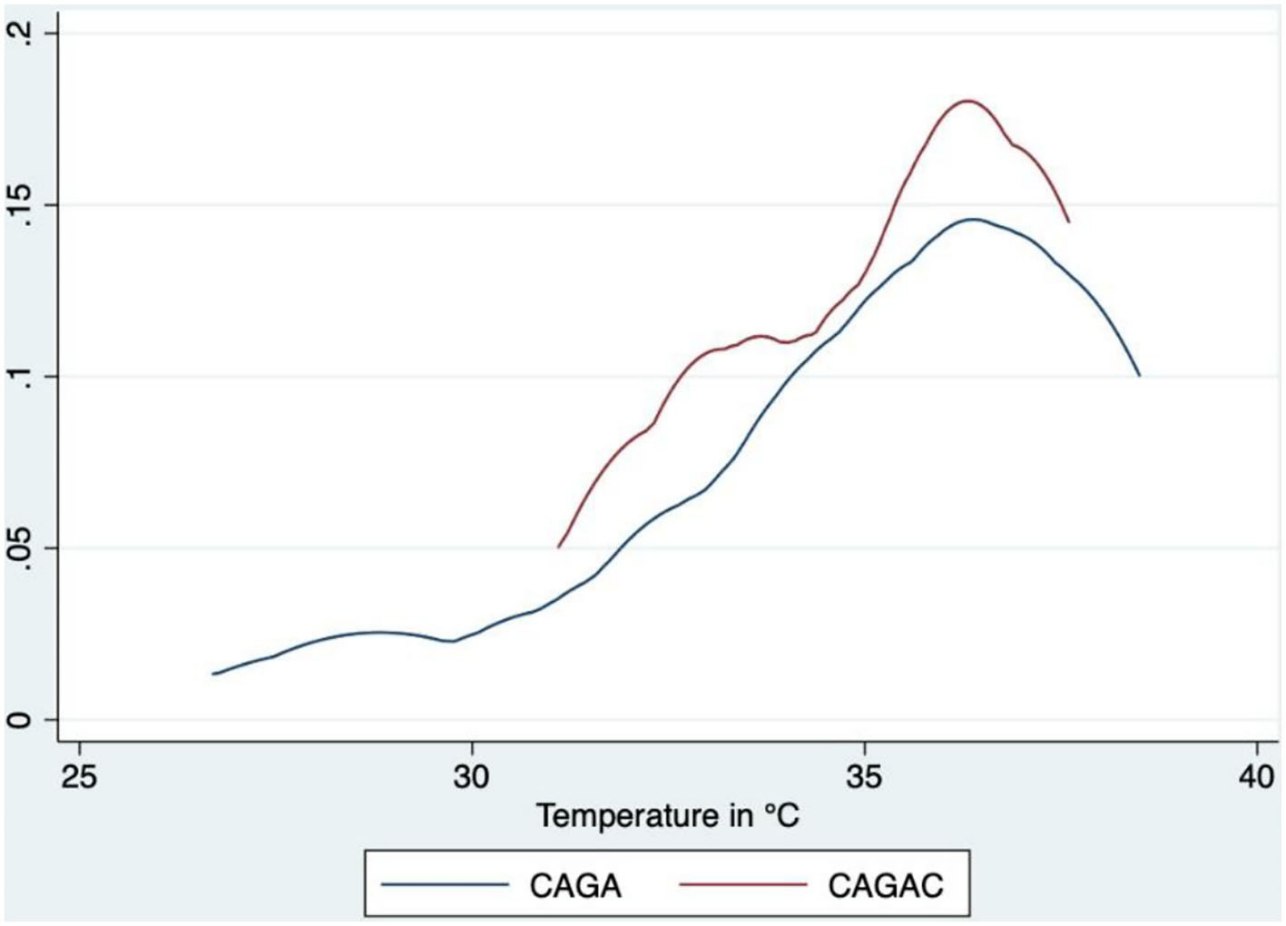
Comparative analysis of probability density functions—SpT. CAGA, circumanal gland adenoma; CAGAC, circumanal gland adenocarcinoma.

As shown in Figures 2, 3, probability density function analysis revealed that CAGACs had a tendency of a higher concentration of lower values. The respective CAGAC curves were located above and to the left of the CAGA curves; specifically, both tumors were colder than healthy sphincter skin. However, a considerable number of CAGACs were colder than CAGAs, which is suggestive of a potential malignancy indicator.

Table 2 shows the probit model estimates, including the marginal effects. Temperature differences ≥ 1°C between tumoral and non-tumoral areas were associated with higher (17.45%) chances of CAGAC diagnosis. There were no significant temperature differences between tumoral and non-tumoral spots.

**Table 2:** Marginal effects (°C) for tumor type identification.

**CAGAC**	
β_1_	−0.107 (0.067)
β_2_	0.1745 (0.087)**
N. of obs. = 51	
Pseudo-*R*^2^ = 0.06	
**Level of sig. 0.05	

β_1_ and β_2_ measure the impact of SpNT−SpT and NTA−TA, respectively, on the likelihood of being an adenocarcinoma, N. of obs. – Number of observations. **P-level of sig. 0.05.

## Discussion

We conducted thermal imaging assessments of dogs, following the guidelines described by Loughin and Marino (25). We performed image acquisition in a closed air-conditioned environment, without windows or air drafts and within 15 min of hair clipping to achieve pre-imaging thermal equilibrium to prevent interference caused by environmental factors (29).

Thermal imaging is a non-invasive and rapid diagnostic method. Furthermore, it is safer than radiography, computed tomography, and magnetic resonance imaging since it does not involve exposure to ionizing radiation (15). The routine use of thermal imaging in clinical practice is facilitated by its ease of use, completion of assessment within seconds, and low cost (34).

In our study, SpT and TA temperatures measured in CAGAs and CAGACs were lower than those in adjacent healthy sphincter skin, which is consistent with findings reported by Xie et al. (12) and Konerding et al. (13).

The ineffectiveness of newly formed vessels could lead to reduced blood flow compared with that in healthy tissues. The anomalous morphology of these new vessels may lead to increased blood viscosity due to the accumulation of erythrocyte sediments and platelet aggregates, which hinders blood flow (14). Although numerous tumors, including those in the breast, have a high blood flow, other tumors have lower mean perfusion rates than those in normal tissue (14). Therefore, the skin surface in the tumor area of perianal tumors could have reduced temperature. However, further research is required regarding the vascular characterization of these tumors.

Another possibility to be explored is the common occurrence of intratumoral necrosis in perianal tumors (5), which may contribute to decreased tumor temperatures. In our study, the pathologist-in-charge did not perform a detailed examination regarding tumor necrosis, including grading and frequency. However, based on the histopathological reports, necrosis was observed in 11 animals, which mostly showed CAGACs, with only one showing adenoma. Future studies considering the frequency and grading of intratumoral necrosis are warranted.

Further, abundant vascularization of the healthy external anal sphincter could lead to it being naturally warmer compared with skin from other regions. Melo et al. (35) analyzed 63 thermographic measurements of healthy skin areas in dogs and compared them with those of mast cell tumors, yielding an average temperature of 35.16°C. In our study, non-tumor sphincters had a mean temperature of 36.49°C; accordingly, sphincters were warmer than healthy skin (*p* < 0.001). Another study measured the body temperature of 47 Greyhounds and analyzed four different points on the right and left pelvic limbs. The observed mean temperature was lower than that for the perianal region in our study (36). Taken together, these findings suggest that healthy anal sphincters are warmer than the standard skin mean. Accordingly, this natural temperature increase in the perianal region contributed to the lower temperature of the CAGA and CAGAC compared with the healthy skin perianal area. However, further studies are needed to characterize the temperature in each region.

Our study demonstrated that thermography is a good complementary diagnostic method for CAGA and CAGAC tumors since it could identify temperature differences between the tumoral and non-tumor areas, which is consistent with findings reported by Pavelski et al. (31) and Melo et al. (29).

Regarding the between-tumor temperature comparison, there were a considerable number of CAGACs that were cooler than CAGAs. There was a 17.45% increase in the probability of CAGAC diagnosis when the temperature difference compared with healthy skin was ≥ 1°C. This indicates that the distance between the tumor and the healthy sphincter is directly associated with the probability of CAGAC diagnosis. Additionally, we found that thermographic examination had 66.67% effectiveness in terms of tumor differentiation.

Previous studies have shown that cytological examination, which is widely used as a diagnostic method in perianal tumors, has greater efficacy than thermography (90 vs. 84.37%, respectively) (37, 38). Although cytology is more effective, it is a more invasive examination; moreover, it cannot immediately yield findings. On the other hand, thermographic examination can be used in conjunction with anamnesis and physical examination at the time of care, which improves the chances of correct diagnosis and therapeutic planning.

## Conclusion

Thermal imaging allows differentiation between healthy and diseased tissues in dogs with perianal tumors; therefore, it may be considered as a good adjuvant diagnostic modality. Our findings suggest that the temperature differences between tumors and healthy sphincter skin are directly correlated with the chances of CAGAC diagnosis.

## Data Availability Statement

The raw data supporting the conclusions of this article will be made available by the authors, without undue reservation.

## Ethics Statement

The animal study was reviewed and approved by Ethic Committee on Animal Use of the School of Veterinary Medicine and Animal Science (University of São Paulo) (CEUA/FMVZ). Written informed consent was obtained from the owners for the participation of their animals in this study.

## Author Contributions

EZ and JM contributed to conception and design of the study. JM guided the research. GF performed the statistical analysis. EZ organized the database and wrote the manuscript. SM and EJ contributed by assisting EZ in surgeries and clinical care, in addition to helping in the discussion. All authors contributed to manuscript revision, read, and approved the submitted version.

## Conflict of Interest

The authors declare that the research was conducted in the absence of any commercial or financial relationships that could be construed as a potential conflict of interest.

## Publisher's Note

All claims expressed in this article are solely those of the authors and do not necessarily represent those of their affiliated organizations, or those of the publisher, the editors and the reviewers. Any product that may be evaluated in this article, or claim that may be made by its manufacturer, is not guaranteed or endorsed by the publisher.
